# Increasing dietary nitrate has no effect on cancellous bone loss or fecal microbiome in ovariectomized rats

**DOI:** 10.1002/mnfr.201600372

**Published:** 2017-03-30

**Authors:** Melissa N. Conley, Cooper Roberts, Thomas J. Sharpton, Urszula T. Iwaniec, Norman G. Hord

**Affiliations:** ^1^ School of Biological and Population Health SciencesCollege of Public Health and Human SciencesOregon State UniversityCorvallisORUSA; ^2^ Center for Healthy Aging ResearchOregon State UniversityCorvallisORUSA; ^3^ Departments of Microbiology and StatisticsCollege of ScienceOregon State UniversityCorvallisORUSA; ^4^ Skeletal Biology LaboratorySchool of Biological and Population Health SciencesOregon State UniversityCorvallisORUSA

**Keywords:** Dietary nitrate, Nitric oxide, Osteoporosis, Postmenopause, Vegetables

## Abstract

**Scope:**

Studies suggest diets rich in fruit and vegetables reduce bone loss, although the specific compounds responsible are unknown. Substrates for endogenous nitric oxide (NO) production, including organic nitrates and dietary nitrate, may support NO production in age‐related conditions, including osteoporosis. We investigated the capability of dietary nitrate to improve NO bioavailability, reduce bone turnover and loss.

**Methods and results:**

Six‐month‐old Sprague Dawley rats [30 ovariectomized (OVX) and 10 sham‐operated (sham)] were randomized into three groups: (i) vehicle (water) control, (ii) low‐dose nitrate (LDN, 0.1 mmol nitrate/kg bw/day), or (iii) high‐dose nitrate (HDN, 1.0 mmol nitrate/kg bw/day) for three weeks. The sham received vehicle. Serum bone turnover markers; bone mass, mineral density, and quality; histomorphometric parameters; and fecal microbiome were examined. Three weeks of LDN or HDN improved NO bioavailability in a dose‐dependent manner. OVX resulted in cancellous bone loss, increased bone turnover, and fecal microbiome changes. OVX increased relative abundances of Firmicutes and decreased Bacteroideceae and Alcaligenaceae. Nitrate did not affect the skeleton or fecal microbiome.

**Conclusion:**

These data indicate that OVX affects the fecal microbiome and that the gut microbiome is associated with bone mass. Three weeks of nitrate supplementation does not slow bone loss or alter the fecal microbiome in OVX.

**Abbreviations**
BMC: bone mineral content
BMD: bone mineral density 2
BV/TV: bone volume/tissue volume
CTx: collagen type 1 cross‐linked C‐telopeptide
DXA: dual‐energy X‐ray absorptiometry
eNOS: endothelial nitric oxide synthase
HDN: high‐dose nitrate
LDN: low‐dose nitrate
NO: nitric oxide 1
NTG: nitroglycerin 2
OVX: ovariectomized 3
WAT: white adipose tissue
μCT: microcomputed tomography

## 1 Introduction

Osteoporosis is a common metabolic bone disease, affecting a third of women and a fifth of men over the age of 65 years 1. In the United States, associated annual health care costs are estimated to be over $20 billion. Menopause‐induced estrogen deficiency causes an increase in bone turnover 2. Increased bone resorption relative to bone formation contributes to reduced bone quality and bone mineral density (BMD), and low BMD is associated with increased fracture risk in postmenopausal women 2, 3.

Some observational studies report that fruit and vegetable consumption is associated with increased bone mineral content (BMC) and BMD 4, 5, 6, 7, 8. Nitrates, present in high concentrations in leafy green and root vegetables, may serve as a dietary component that supports bone health. Since dietary nitrate enhances NO bioavailability in a dose‐dependent fashion 9, this dietary compound may be a viable option for reducing bone loss. Vegetable intake accounts for ∼80% of dietary nitrate consumption in human diets 10; dietary nitrates can be reduced to nitric oxide (NO) via non‐enzymatic reduction by lingual bacteria and a variety of mammalian reductases and increase NO bioavailability through the nitrate‐nitrite‐NO pathway 11, 12. Gastric metabolism of nitrate and nitrite is associated with both health benefits and risks 13, including the formation of potentially carcinogenic nitrosamines promoted by nitrites in processed meats which can be inhibited by flavonoids and other compounds in vegetables 14. Importantly, both the European Food Standards Agency (EFSA) and a recent NHLBI panel concluded that current research does not support a role for nitrate consumed as vegetables as a carcinogen 10.

NO plays extensive roles in physiological processes, ranging from vascular homeostasis to host defense, cellular energetics, and nerve transmission 15. There are two major sources of endogenous NO in the body: the nitric oxide synthase (NOS) system that uses L‐arginine as a substrate and the mammalian nitrate‐nitrite‐NO pathway that uses dietary nitrate and nitrate derived from oxidation of NOS‐derived NO. Under normal conditions, these contribute about equally to NO homeostasis 16. The mammalian nitrate‐nitrite‐NO pathway is involved in the regulation of blood flow and blood pressure, cell signaling, and tissue responses to hypoxia 17. During aging and hypoxia/ischemia, the highly oxygen‐dependent NOSs become less effective in generating NO, in contrast to the nitrate‐nitrite‐NO pathway, in which NO formation from nitrite reduction in tissues is enhanced during hypoxia and low pH 18. Aging is associated with decreased endothelial NOS‐dependent NO synthesis and endothelium‐dependent vasodilation, suggesting a possible role for dietary nitrates to support NO production in age‐related conditions like osteoporosis. 19, 20.

Organic nitrates (e.g., nitroglycerin [NTG]) have been used to treat cardiovascular disorders for over 100 years 21. Postmenopausal women using organic nitrates had improved BMD compared to non‐nitrate users 20. Studies in an ovariectomized (OVX) animal model of bone loss demonstrated increased NO bioavailability using NTG as an NO donor and improved BMD and decreased bone turnover 22, 23, 24. While pilot studies in humans suggested a beneficial effect, the Phase III NOVEL trial found no significant effect of NTG on lumbar spine BMD after two years of intervention 25, 26, 27.

The gut microbiome may also affect bone mass 28 through the production of NO in the gut 29. Several investigations have shown the gut microbiome plays a role in regulating bone mass suggesting interactions between diet and the gut microbiome could have important consequences for bone loss 28, 30, 31. Since we hypothesized that fecal microbiome composition is associated with dietary nitrate status and bone loss, we interrogated the dietary nitrate‐dependent changes in the fecal microbiome. The objectives of this study were to quantify the ability of dietary nitrate to improve NO bioavailability, reduce bone turnover and loss, as well as alter the fecal microbiome composition using an OVX rat model.

## 2 Materials and methods

### 2.1 Experimental design

A total of 40 six‐month‐old female Sprague Dawley rats [30 ovariectomized (OVX) and 10 sham‐operated (sham) at 5.5 month of age] were purchased from Charles River Laboratory (Hollister, California). Three days after arrival at Oregon State University OVX rats were randomized by weight into 1 of 3 treatment groups (*n* = 10 per group): (i) vehicle (water) control, (ii) low‐dose nitrate (LDN, 0.1 mmol nitrate/kg bw/day), or (iii) high‐dose nitrate (HDN, 1.0 mmol nitrate/kg bw/day). The sham controls received vehicle. At study initiation, sodium nitrate was added to water at 0.14 or 1.4 g/L to achieve 0.1 or 1.0 mmol nitrate intake per kilogram body weight per day; the concentration of nitrate was adjusted during body weight gain to assure consistent dietary nitrate concentration throughout the study. Food (TD.2018 chow, Teklad Lab Animal Diets, 118/5.4 nmol of nitrate/nitrite per g) was provided *ad libitum* to all animals. The rats were single‐housed and maintained on a 12‐hour light:12‐hour dark cycle for the three‐week duration of study. The Institutional Animal Care and Use Committee at Oregon State University approved the experimental protocol under ACUP 4532. Animals were maintained in accordance with the NIH Guide for the Care and Use of Laboratory Animals.

Food and water consumption and body weight were measured twice a week. These data were used for adjusting nitrate levels in the water to maintain appropriate nitrate concentrations throughout the study. Water was replaced every other day for the duration of experiment. The fluorochrome calcein (20  mg/kg, Sigma‐Aldrich, St. Louis, MO), was injected subcutaneously 9 and 2 days before sacrifice to label mineralizing bone. Fecal samples were collected at the end of the three‐week study and stored at –80°C. For tissue collection all rats were fasted overnight, then anesthetized with 2–3% isoflurane delivered in oxygen, and death was induced by exsanguination from the heart. Serum and blood were collected and stored at −80 °C for measurement of serum global markers of bone turnover and blood nitrate and nitrite levels. Uteri and abdominal white adipose tissue (WAT) were excised and weighed. Tibiae were removed and stored in 70% ethanol for analysis using dual‐energy X‐ray absorptiometry (DXA), microcomputed tomography (μCT), and histomorphometry.

### 2.2 Quantification of blood nitrate and nitrite levels

#### 2.2.1 Blood collection and pretreatment

Whole blood was obtained by cardiac puncture and pretreated for nitrite and nitrate analysis, as described previously 32. Briefly, heparinized whole blood was mixed with nitrite‐preserving solution [K_3_Fe(CN)_6_, N‐ethylmaleimide, water, Nonidet P‐40] and kept frozen at −80°C until analysis. For nitrate analysis, heparinized whole blood was mixed with deionized water at a 1∶9 ratio between the blood and water and kept frozen at −80°C until analysis.

#### 2.2.2 Nitrate and nitrite analysis

Nitrate and nitrite content was analyzed using a standard gas phase chemiluminescence method (NO Analyzer (NOA), model 280i, GE Analytical Instruments, Boulder, CO) with helium as carrier gas, as described elsewhere 32. Briefly, for nitrate analysis, samples were injected into purge vessel of the NO analyzer containing 7 mL of heated (95°C) vanadium chloride (0.8 g of vanadium chloride dissolved in 100 mL of 1M hydrochloric acid) solution. For nitrite analysis, samples were deproteinized with methanol, centrifuged for 3 min at 15 000 rpm, and then supernatants were injected into the purge vessel of the NOA containing 7 mL of tri‐iodide (1 g potassium iodide, 0.65 g iodine, 20 mL water and 70 mL glacial acetic acid) solution. The concentration of nitrate and nitrite in analyzed samples was deduced from standard concentration vs. peak area curves constructed with sodium nitrate (10 μM) and sodium nitrite (1 μM), respectively. A correction for the nitrite concentration in nitrite‐preserving solution and methanol, nitrate concentration in deionized water, and molar mass of sodium was made when calculating levels of nitrate and nitrite ions in the samples analyzed.

Average daily nitrate intake from food was derived from average daily food intake and quantification of dietary nitrate present in TD.2018 diet, as described in the methods section. Average daily nitrate intake from water was derived from average daily water intake and sodium nitrate concentrations in water provided. Total average daily nitrate intake was determined by totaling average daily nitrate intake from food and water.

### 2.3 Serum markers of bone turnover

Serum osteocalcin was measured using a rat Gla‐osteocalcin High Sensitive EIA kit obtained from Clontech Takara. Serum C‐terminal telopeptide (CTx) was measured using a rat CTx ELISA kit from Life Sciences Advanced Technologies (St. Petersburg, FL).

### 2.4 Dual‐energy X‐ray absorptiometry

Tibial bone mineral content (BMC; mg) and area (cm^2^) were measured ex vivo using dual‐energy x‐ray absorptiometry (DXA; Piximus; Lunar Corp., Madison, WI). Bone mineral density (BMD) was calculated as BMC per area (mg/cm^2^).

### 2.5 Microcomputed tomography

Nondestructive three‐dimensional evaluation of bone microarchitecture was completed using μCT. Tibiae were scanned at a voxel size of 16×16×16 μm (55 kV_p_ x‐ray voltage, 145 μA intensity, and 200 ms integration time) using a Scanco μCT40 scanner (Scanco Medical AG, Basserdorf, Switzerland). Filtering parameters sigma and support were set to 0.8 and 1, respectively. Threshold for analysis was determined empirically and set at 245 (range, 0–1000) for both cancellous bone and cortical bone. Cancellous bone was assessed in 94 slices (1504 μm) (1504 μm distal to the growth plate) and 33 ± 1 slices (528 ± 16μm) in the proximal tibia metaphysis and proximal tibia epiphysis, respectively (Supporting Information Fig. 1: VOIs). Cortical bone was assessed at 62 slices (992 μm) distal to the midshaft. Midshaft was defined as midpoint between the top and bottom of each tibia. Direct cancellous bone measurements included cancellous bone volume fraction (bone volume/tissue volume, BV/TV; volume of tissue occupied by cancellous bone, %), trabecular number (number of trabecular intercepts, mm^−1^), trabecular thickness (mean thickness of individual trabeculae, um), and trabecular separation (mean distance between trabeculae, um). Direct cortical measurements included cross‐sectional volume (volume of cortical bone and bone marrow, mm^3^), cortical volume (mm^3^), marrow volume (mm^3^), and cortical thickness (μm).

### 2.6 Histomorphometry

Histological methods applied here have been previously described 33 and are summarized in Supporting Information File 1. Histomorphometric data were collected using the OsteoMeasure System (OsteoMetrics, Inc., Atlanta, GA, USA) and are reported using standard two‐dimensional nomenclature (Dempster, 2012).

### 2.7 Fecal DNA isolation and 16S amplicon sequencing

Samples were processed as previously described 34. Fecal DNA was isolated using QIAamp DNA stool mini‐kits (Qiagen, Valencia, CA) per manufacturer's instructions. 16S rRNA PCR amplification was conducted according to established methods 35. Briefly, each sample's extracted DNA was subjected to PCR reactions to amplify the V4 region of the 16S locus using PCR primers (515F and 806R) that include Illumina adapters and sample‐specific barcodes 35. PCR amplicons from individual rat samples were cleaned using the Qiagen QIAquick PCR cleanup kit (Germantown, MD) and pooled. An aliquot of the pooled 16S library was sequenced on an Illumina MiSeq (v3 chemistry) at the Center for Genome Research and Biocomputing core facility (Oregon State University, OR). This generated ∼596 thousand 300 bp single end reads (median reads per sample = 15025).

### 2.8 Rat Osteoporosis RT² Profiler PCR Array

We did not observe a beneficial effect of dietary nitrate on the skeleton of OVX rats. However, it is possible that a normal diet provided sufficient levels of nitrate. We therefore evaluated the effects of nitrate deficiency and supplementation on expression of genes related to bone formation and resorption. Growing rats were studied based on the expectation that the growing skeleton would be especially sensitive to nitrate levels. We found no significant changes in gene expression in response to either dietary nitrate or nitrite treatment (data not shown). Associated methods and analyses are described in detail in Supporting Information File 1.

### 2.9 Bioinformatics and statistical analysis

Mean responses of individual variables were compared between sham, OVX vehicle, LDN, and HDN groups using separate one‐way analyses of variance (ANOVA). A modified F test was used when the assumption of equal variance was violated, with Welch's two‐sample *t*‐test used for pairwise comparisons 36. The Kruskal–Wallis nonparametric test was used when only the normality assumption was violated, in which case the Wilcoxon—Mann–Whitney test was used for pairwise comparisons. The Benjamini and Hochberg method for maintaining the false discovery rate at 5% was used to adjust for multiple comparisons 37. Differences were considered significant at *p* <0.05. All data are expressed as mean ± SE. Data analysis was performed using R version 2.12.

Bioinformatic analyses were performed as previously described 34. Samples were rarefied to 12 000 reads, and alpha‐ (i.e., richness) and beta‐diversity (i.e., weighted and unweighted UniFrac distances) were subsequently quantified using the core_diversity_analyses.py script in QIIME 38. Statistical analyses were conducted in R. The coin package was used to implement robust statistical tests and identify differences in the gut microbiome communities of sham and OVX rats (i.e., Wilcoxon tests). For OTU level analyses, OTUs were filtered based on presence in three or more samples. False discovery rates were quantified using Bonferroni corrections for phylum level tests, and the *q*‐value software package was used for remaining comparisons at the taxonomic level. The Kendall package was used to quantify co‐variation between host parameters and OTU abundance (i.e. Kendall’ tau).

## 3 Results

### 3.1 Dietary nitrate intakes

Average daily nitrate intakes for each group are shown in Table 1. There were no differences in average daily nitrate intake from food between sham, OVX vehicle, LDN, or HDN groups. Total daily nitrate intakes were not significantly different between OVX and sham, while LDN and HDN groups had significantly higher total nitrate intake compared to OVX vehicle.

**Table 1:** Average body weight, food and water intake, daily nitrate intake, uterine weights, and abdominal WAT across each group

Endpoint	Sham‐Operated	Ovariectomized			FDR‐adjusted *p*‐value comparing the four groups
	Control (*n* = 10)	Control (*n* = 10)	Low NO3 (*n* = 10)	High NO3 (n = 10)	
Body weight(g)	314.3 ± 5.9	370.1 ± 10.38^a^	368.6 ± 9.94^a^	365.8 ± 9.97^a^	0.004
Average daily food intake (g/d)	16.24 ± 0.43	18.97 ± 0.71	18.77 ± 0.65	17.91 ± 0.45	0.110
Average daily water intake (mL/g)	22.48 ± 1.77	17.16 ± 0.95	18.73 ± 1.36	21.29 ± 1.15	0.398
Average daily nitrate from food (μM/d)	2.35 ± 0.06	2.75 ± 0.1	2.72 ± 0.09	2.5 ± 0.06	0.110
Average daily nitrate from water (μM/d)	– *±* –	– *±* –	30.85 *±* 2.24	350.67 *±* 18.91^c^	N/A
Average total daily nitrate intake (μM/d)	2.35 ± 0.06	2.75 ± 0.1	33.57 ± 2.29^b^	353.27 ± 18.91^b^, ^c^	0.003
Uterine weight(g)	0.728 ± 0.060	0.157 ± 0.01^a^	0.155 ± 0.01^a^	0.1612 ± 0.01^a^	0.003
Abdominal white adipose tissue weight (g)	9.37 ± 0.54	13.22 ± 1.06	14.15 ± 1.44	13.39 ± 0.51	0.096

Data are mean ± SE

a Different than sham, *p* < 0.05.

b Different than OVX control, *p* < 0.05.

c Different than low NO3, *p* < 0.05.

*The Benjamini–Hochberg method for maintaining the family‐wise error rate at 5% was used to adjust for multiple comparisons

Food intake, water intake, and body weights were collected twice per week for duration of the study.

These data were used to calculcate averages.

Average total daily nitrate intake quantified by totaling the average daily nitrate from food and water.

### 3.2 Effects of dietary nitrate on blood nitrate and nitrite levels

**Figure 1:**
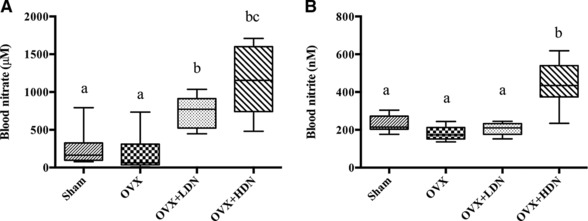
**(**a) OVX had no effect on blood nitrate levels and increasing dietary nitrate resulted in increased blood nitrate levels and (b) OVX had no effect on blood nitrite levels compared to sham, while significantly increased blood nitrite levels were observed in the OVX‐HDN treatment group. OVX, ovariectomized; LDN, low‐dose nitrate 0.1 mmol sodium nitrate kg/day; and HDN, high‐dose nitrate 1 mmol sodium nitrate kg/day. Data represent means ±SE (*n* = 10 rats/group). ^*^Differences were considered significant at Benjamini‐Hochberg adjusted *p* < 0.05; Groups not sharing a superscript are significantly different.

The effects of OVX and dietary nitrate supplementation on blood nitrate and blood nitrite levels are summarized in Fig. 1. There were no significant differences in blood nitrate levels between OVX vehicle and sham controls (Fig. 1A). The LDN and HDN groups had significant increases in blood nitrate compared to OVX vehicle. There were also no significant differences in blood nitrite levels between OVX vehicle and shams (Fig. 1B). There were no significant differences in blood nitrite levels in LDN group compared to vehicle, while the HDN group had significantly higher blood nitrite levels compared to OVX vehicle and LDN groups.

### 3.3 Effects of dietary nitrate on food and water intake, body weight, uterine weight, and abdominal white adipose tissue weight

Average daily food and water intake, body weights, uterine weights, and abdominal white adipose tissue (WAT) weights for each group are shown in Table 1. All OVX groups had significantly increased body weight and decreased uterine weight compared to sham controls with no differences observed between OVX, LDN, and HDN groups. There were no significant differences in food intake between OVX and sham, or OVX vehicle, LDN, and HDN groups. There were no observed differences in WAT weights between groups.

### 3.4 Effects of ovariectomy on bone

The effects of OVX on bone mass and architecture are summarized in Table 2. Significant differences in tibial bone area, BMC, or BMD were not detected between OVX and sham controls (Table 2). In the proximal tibia metaphysis, OVX resulted in significantly lower BV/TV, trabecular number, and trabecular separation and no differences in trabecular thickness. In the proximal tibia epiphysis, OVX resulted in significantly lower BV/TV and no significant differences in OVX on trabecular number trabecular separation, or trabecular thickness compared to sham. Significant differences in cortical endpoints (cross‐sectional area, cortical area, marrow area, or cortical thickness) were not detected between OVX and sham.

**Table 2:** Dietary nitrate had no effects on BMC and BMD in total tibia, on cancellous bone in the proximal tibial metaphysis or proximal tibial epiphysis and on cortical bone in the tibial diaphysis in ovariectomized Sprague Dawley rats

Sham‐Operated	Ovariectomized				
Endpoint	Control (*n* = 10)	Control (*n* = 10)	Low NO3 (*n* = 10)	High NO3 (*n* = 10)	FDR‐adjusted *p*‐value comparing the four groups
Total Tibia					
Bone Area (cm^2^)	2.40 ± 0.05	2.62 ± 0.06	2.55 ± 0.03	2.56 ± 0.03	0.172
BMC (g)	0.364 ± 0.009	0.373 ± 0.015	0.361 ± 0.008	0.366 ± 0.010	1.000
BMD (g/cm^2^)	0.152 ± 0.005	0.142 ± 0.003	0.142 ± 0.002	0.143 ± 0.003	1.000
microComputed Tomography					
Proximal Tibia Epiphysis (cancellous bone)					
Bone Volume/Tissue Volume (%)	43.52 ± 1.47	37.14 ± 1.12^a^	38.42 ± 1.12^a^	37.50 ± 1.15^a^	0.041
Trabecular Number (1/mm)	5.13 ± 0.19	4.54 ± 0.11	4.69 ± 0.12	4.69 ± 0.18	0.540
Trabecular Thickness (μm)	98 ± 3	95 ± 2	95 ± 2	94 ± 2	1.000
Trabecular Spacing (μm)	182 ± 6	212 ± 6	202 ± 5	208 ± 7	0.088
Proximal Tibia Metaphysis (cancellous bone)					
Bone Volume/Tissue Volume (%)	28.95 *±* 3.22	14.14 *±* 1.09^a^	14.77 *±* 1.72^a^	14.31 *±* 1.24^a^	0.003
Trabecular Number (1/mm)	5.44 *±* 0.26	3.93 *±* 0.22^a^	3.97 *±* 0.15^a^	3.78 *±* 0.2^a^	0.003
Trabecular Thickness (μm)	71 *±* 3	64 *±* 2	63 *±* 2	63 *±* 1	0.446
Trabecular Spacing (μm)	168 *±* 10	252 *±* 16^a^	244 *±* 10^a^	261 *±* 15^a^	0.003
Midshaft Tibia (cortical bone)					
Cross‐Sectional Volume (mm³)	5.96 *±* 0.19	6.43 *±* 0.26	6.05 *±* 0.15	6.19 *±* 0.14	1.000
Cortical Volume (mm³)	4.56 *±* 0.14	4.93 *±* 0.17	4.73 *±* 0.12	4.78 *±* 0.11	1.000
Marrow Volume (mm³)	1.40 *±* 0.07	1.50 *±* 0.10	1.32 *±* 0.04	1.41 *±* 0.06	1.000
Cortical Thickness (μm)	654 *±* 13	676 *±* 10	677 *±* 14	673 *±* 11	1.000
I_polar_ (mm⁴)	5.96 *±* 0.37	7.06 *±* 0.55	6.20 *±* 0.29	6.44 *±* 0.29	1.000

Data are mean ± SE

a Different than sham, p < 0.05

Different than OVX control, p < 0.05

Different than low NO3, p < 0.05

*The Benjamini–Hochberg method for maintaining the family‐wise error rate at 5% was used to adjust for multiple comparisons

BMC, bone mineral content; BMD, bone mineral density

Rats were ovariectomized at 6 months of age and treated with one of two doses of nitrate for three weeks.

**Figure 2:**
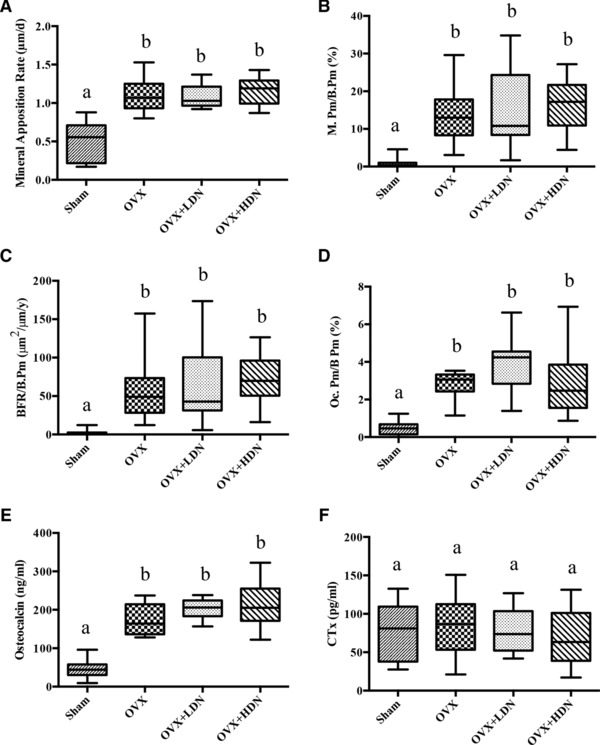
**(**a) Dietary nitrate had no effect on (a) mineral apposition rate (MAR), (b) mineralizing perimeter (mineralizing bone/bone perimeter, M.Pm/B.Pm), (c) bone formation rate (bone formation rate /bone perimeter, BFR/B.Pm), or (d) osteoclast perimeter (osteoclast perimeter /bone perimeter, Oc.Pm/B.Pm) in the proximal tibia metaphysis of ovariectomized Sprague Dawley rats as determined by histology; Dietary nitrate had no effect on serum biochemical markers of (e) bone formation (Osteocalcin), or f) bone resorption (CTx). OVX, ovariectomized; LDN, low‐dose nitrate 0.1 mmol sodium nitrate kg/ day; and HDN, high‐dose nitrate 1 mmol sodium nitrate kg/ day. Data represent means ±SE (*n* = 10 rats/group). ^*^Differences were considered significant at Benjamini–Hochberg adjusted *p* < 0.05; Groups not sharing a superscript are significantly different.

Histological analyses of proximal tibia metaphysis revealed the OVX vehicle group had significantly higher mineral apposition rate, mineralizing perimeter, bone formation rate, and osteoclast perimeter compared to sham controls (Fig. 2A–D). OVX vehicle had significantly higher osteocalcin levels compared to sham controls (Fig. 2E). No significant differences in CTx levels between OVX vehicle and sham were observed (Fig. 2F)

### 3.5 Effects of dietary nitrate supplementation on bone in ovariectomized rats

The effects of dietary nitrate supplementation on bone are summarized in Table 2. Significant differences in tibial BMC, bone area, or BMD were not detected between OVX rats treated with vehicle, LDN, or HDN. Significant differences in cancellous bone (BV/TV, trabecular number, trabecular thickness, and trabecular separation) were not observed among treatment groups. Similarly, significant differences in cortical endpoints (cross‐sectional volume, cortical volume, marrow volume, or cortical thickness) were not detected among OVX rats treated with vehicle, LDN, or HDN. Histological analyses of proximal tibia metaphysis revealed there were no significant differences in mineral apposition rate, mineralizing perimeter, or bone formation rate between OVX vehicle, LDN, or HDN groups (Fig. 2A–D). Significant differences in cortical endpoints (cross‐sectional area, cortical area, marrow area, or cortical thickness) were not detected between OVX, LDN, and HDN groups.

### 3.6 Effects of dietary nitrate supplementation on serum biochemical markers of bone turnover

**Figure 3:**
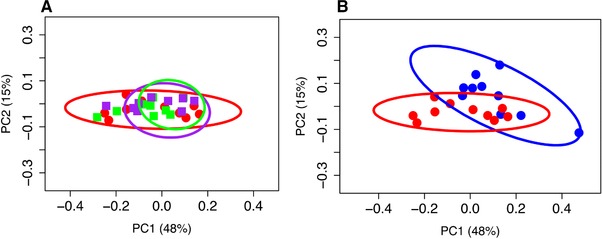
**(**a) Principal coordinates analysis using weighted UniFrac distance on 16S sequences from fecal microbiota of sham (blue) and OVX (red) rats showing there are distinct phylogenetic differences in the gut microbiome between sham and OVX rats (Adonis, *R* ^2^ = 0.17, *p* = 0.017); (b) Principal coordinates analysis using weighted UniFrac distance on 16S sequences from fecal microbiota of OVX (red), low (purple) and high (green) dose nitrate treated rats shows no distinct phylogenetic differences in the gut microbiome between OVX controls and OVX rats supplemented with nitrate. These results are consistent at the taxonomic level. Points represent individual rats. Ellipses represent 95% confidence intervals.

The effects of OVX and dietary nitrate supplementation on serum biochemical markers of bone resorption (CTx) and bone formation (osteocalcin) are show in Fig. 3E and F. There were no significant differences in osteocalcin or CTx levels between OVX vehicle group, LDN, or HDN.

### 3.7 Effects of ovariectomy and dietary nitrate supplementation on fecal microbiota composition

In order to identify possible gut microbiota signatures with OVX or dietary nitrate, we compared the fecal microbiota composition of sham rats to OVX rats treated with vehicle, LDN, or HDN. We first assessed the intersample diversity between the sham and OVX rats. We used UniFrac, which normalizes intersample taxonomic differences by the phylogenetic diversity of the microbial lineages observed in the samples (i.e., samples containing more phylogenetically similar taxa produce a relatively lower distance). A principal coordinates analysis (PCoA) of fecal samples based on their weighted UniFrac distances reveals that samples weakly but significantly cluster by OVX status, as supported by a permutational multivariate analysis of variance (*R* ^2^ = 0.17, *p* = 0.017) (Fig. 3B). This finding suggests the sham and OVX rats exhibit microbial communities with different evolutionary histories. These results are qualitatively consistent with those obtained using unweighted UniFrac and indicate that there are distinct phylogenetic differences in the gut microbiome between sham and OVX rats. We also observe significant differences between within‐group and between group beta diversity between sham and OVX (Supporting Information Fig. 2). However, we did not observe any clustering between OVX vehicle, LDN, or HDN groups based on UniFrac distances (Fig. 3A), or significant differences between within‐group and between‐group beta‐diversity variability across these groups (Supporting Information Fig. 2).

We then explored the structure of these communities at various taxonomic levels to understand potential taxonomic signatures that may stratify samples based on OVX status. Using 16S rRNA ribosomal genes as a marker, sequences were clustered into operational taxonomic units (OTUs) using a threshold of 97% sequence similarity from the GreenGenes database 39 using the QIIME open‐reference OTU‐picking protocol. OTUs were then taxonomically annotated using UCLUST. As expected, given prior characterizations of the rodent gut microbiome, all rats were dominated by the phyla Bacteroidetes and Firmicutes 40, 41. We found a trend of increases in Proteobacteria abundance in the OVX rats (Bonferroni adjusted *p* = 0.052), whereas Firmicutes were more abundant in the sham rats (Bonferroni adjusted *p* = 0.015). We also observed a non‐significant increase in the abundance of Bacteroidetes in OVX animals (Bonferroni adjusted *p* = 0.12). At the family level, Bacteroidaceae, Erysipelotrichaceae, and Alcaligenaceae were lower in OVX (*q* < 0.20). The genera *Bacteroides* and *Sutterella* were also lower in the OVX rats (*q* < 0.20). Consistent with our analysis at the phylogenetic level, we did not observe any significant differences in fecal microbiome communities between OVX vehicle, LDN, or HDN groups at the taxonomic level. Finally, we were unable to identify any taxa that correlate with blood nitrate or nitrite levels, or bone status including cancellous BV/TV in proximal tibia metaphysis, cortical volume in tibia diaphysis, or total tibia BMD.

## 4 Discussion

The goal of this study was to evaluate the effects of dietary nitrate on bone in a rat model of OVX‐induced bone loss. Three weeks of dietary nitrate supplementation in water at 0.1 mmol nitrate/kg bw/day or 1.0 mmol nitrate/kg bw/day to rats increased blood levels of nitrate in a dose‐dependent fashion and to concentrations comparable to those in humans consuming high‐nitrate fruit and vegetables (e.g., 0.1 mmol/kg/d equates to ∼372 mg and 1.0 mmol/kg/d to 3.72 grams, respectively) 9. Dietary nitrate concentrations of ∼0.1 mmol/kg/d in rats have been demonstrated to lower blood pressure 16, and intakes of ∼372 mg total nitrate per day reverse vascular dysfunction in older adult humans 42. Three weeks of dietary nitrate supplementation, a period adequate to observe rapid OVX‐associated bone loss and treatment‐associated effects 43, had no effect on tibial cancellous or cortical bone mass and architecture or histomorphometric indices of bone formation or resorption in OVX rats. Furthermore, increased dietary nitrate had no significant effect on serum biochemical markers of bone turnover.

We are not aware of prior animal studies investigating dietary nitrate effects on bone mass and turnover. One in vitro study ascribed estrogenic activity to the nitrite anion in a transcription assay 44. The relationship between NO and bone physiology is complex. Osteoclasts and osteoblasts both constitutively express the endothelial isoform of nitric oxide synthase (eNOS), implying a role for NO in bone metabolism. Exogenous nitrates like NTG may influence bone cells indirectly as NO donors. The molecular targets for NO in bone cells are poorly understood, although in vitro work suggests that NO has biphasic effects on both osteoclasts and osteoblasts, demonstrating the ability to stimulate or inhibit cell activity depending on the amount of NO present, as well as which NOS isoform is active 45, 46, 47. Nitrates may also affect osteoclasts and osteoblasts; NO may influence osteoclast activity, in part, via the receptor activator of NF‐kappaB ligand (RANKL)/osteoprotegerin (OPG) pathway. High levels of NO stimulate OPG, which binds to RANKL and prevents the binding of RANKL to the receptor activator of NF‐kappaB (RANK), decreasing osteoclast activity 48. eNOS global knockout animals exhibit lower BMD, bone formation, and osteoblast activity; with little to no effect on bone resorption, suggesting NO may be important for osteoblast function 49, 50. Previous research shows NO synthesis is induced in osteoblasts and osteocytes by mechanical strain and shear stress 51, 52, 53, 54.

Aging is associated with decreased eNOS‐dependent NO synthesis and endothelium‐dependent vasodilation 18. Reduced cofactor availability for eNOS can decrease NO production 19. Previous reports show that OVX significantly lowers plasma NO metabolite [NO_x;_ plasma nitrate+nitrite] levels in rats 55. In postmenopausal women receiving hormone replacement therapy (HRT), plasma NO_x_ levels are positively correlated with estrogen status 56. Thus, age‐associated decreases in NO production may contribute to osteoporosis risk. However, we did not observe a significant decrease in either blood nitrate or nitrite levels in OVX rats.

Studies in OVX rats show organic nitrates slow bone loss by reducing bone turnover 22, 23, 24. Human data on organic nitrate effects on bone are generally concordant with the animal data, but there are conflicting results. A randomized controlled trial (RCT) to study the effects of NTG ointment compared to oral estrogen in oophorectimized women demonstrated that NTG was equivalent to a standard estrogen dose in preserving BMD 25. Furthermore, NTG significantly decreased NTx (bone resorption) and increased serum osteocalcin and serum bone‐specific alkaline phosphatase (bone formation) 25. A follow‐up RCT conducted in postmenopausal women reported equivalent effects of isosorbide mononitrate (20 mg/day) or alendronate (70 mg/week) on lumbar BMD after 12 months of treatment 26, suggesting that organic nitrate is able to increase BMD and slow bone turnover in humans. However, the Phase III clinical trial NOVEL, evaluating NTG on lumbar spine BMD in postmenopausal women, found no significant differences between the NTG and control groups after two years of intervention 27. Poor compliance and suboptimal dose (22.5 mg NTG/day) potentially explain the null findings. Most patients were taking <15 mg of active NTG per day, far below the proposed therapeutic window for bone.

While both organic and inorganic nitrates mediate their principal effects through NO production, there are notable differences. Inorganic nitrates are small, water‐soluble ions present in the diet and produced endogenously by oxidation of NO, while organic nitrates are synthetic and structurally more complex 57. These structural differences, together with pharmacokinetic differences, may contribute to the observed differences in animal studies.

Dietary nitrates rely on lingual reduction of salivary nitrate via enterosalivary circulation 58, to produce nitrite and NO. Biotransformation of organic nitrates is not fully understood and can vary widely with the class of organic nitrate 59. Organic nitrates typically undergo liver first‐pass metabolism; however, the exact mechanism of denitration (release of NO) in the vasculature or different tissue sites remains a matter of debate 60, 61. Organic nitrates have potent acute effects, while the metabolic conversion of inorganic nitrate to nitric oxide is more efficient under hypoxic and acidic conditions that may not have been reproduced under these study conditions 57. Thus, differential metabolism may explain why dietary nitrate does not affect the estrogen‐deficient skeleton in an analogous manner to organic nitrates. The form of supplemented nitrate may alter efficacy. For instance, vegetable juices were more efficacious than sodium nitrate in lowering blood pressure in humans, indicating that other components of vegetables may increase efficacy 62. While we demonstrate that sodium nitrate has no effect on bone when supplemented in water, it remains unknown if nitrate‐rich foods have a beneficial effect on the osteoporotic skeleton. Future studies should consider evaluation of nitrate‐rich foods, supplementation prior to ovarian hormone deficiency, and inclusion of a positive control, such as organic nitrate, to show responsiveness to exogenous substrate for NO production in the animal model.

Salivary nitrate reduction to nitrite by nitrate reductases in lingual bacteria is one of the earliest examples of commensalism 13. Prior work has demonstrated dietary nitrate can alter oral microbiome composition 63, 64. However, little is known about interaction between dietary nitrate and the gut microbiome. Certain probiotic bacterial strains have been shown to generate NO within the gastrointestinal tract, highlighting the physiological context‐dependence of the nitrate‐nitrite‐nitric oxide cycle 29. Given the ability of diet to modulate the gut microbiome, and the gut microbiome's association with bone mass, we evaluated the ability of dietary nitrate to influence fecal microbiome composition. We did not observe significant differences in fecal microbiome composition or diversity between OVX vehicle, LDN, or HDN groups (Fig. 3A). However, variation in cellular abundance of the microbiome could exist. It is also possible that OVX effects overwhelm the effects of the dietary nitrate‐related gut microbiome, or that nitrate is influencing the gut microbiome in a region of the gut that is not well represented by the stool microbiome, as nitrate is largely absorbed in the upper small intestine 11.

We did observe differences in fecal microbiome composition between OVX and sham controls. An accumulating body of literature suggests a role for the gut microbiome in the regulation of bone mass. For instance, germ‐free (GF) mice have significantly increased bone mass compared to conventionally raised mice 65. Further, OVX GF mice are protected from both cancellous and cortical bone loss 30. Since altered microbiome composition is associated with chronic disease and that specific microbiota interacts with the immune system 66, 67, 68, 69, 70, it has been hypothesized that gut microbiome composition may influence the magnitude of bone loss in postmenopausal women 30. Sex steroid deficiency results in a chronic inflammatory state that may contribute to osteoporosis 71. The microbiome's effects on bone mass may be mediated via alteration of the immune system and regulation of osteoclastogenesis, which results in greater bone loss in postmenopause when women lose the immunosuppressive effects of estrogen 30, 72, 73. This evidence suggests a putative role of the gut microbiome in the bone loss observed in sex‐steroid deficiency. Currently, our understanding of OVX‐related changes in the composition of the enteric microbiome in rats is limited to one study. Characterizing this relationship is useful given the growing body of literature that posits a relationship between bone mass and the gut microbiome 28, 30, 31. York et al. characterized fecal microbial composition of low‐ and high‐aerobic capacity rats in relation to OVX 74. They found phylum level differences and a significantly lower Firmicutes:Bacteroidetes (F:B) ratio in OVX rats.

We observed differences in the community structure of the fecal microbiome between sham and OVX rats (Fig. 3B). We also identified specific taxa that stratify OVX and sham animals, and observed increased Firmicutes abundance in OVX compared to sham (*p* = 0.015). In contrast to York et al., we observed a significantly higher F:B ratio associated with OVX (*p* = 0.03). This result indicates that the F:B statistic may not robustly associate with OVX status and may be subject to study effects, as has been observed in microbiome investigations of obesity 75, 76. Future work should consider how specific organisms within the Bacteroidetes and Firmicutes phyla interact with estrogen‐related processes in the gut. York et al. did not report evaluation of phylotype differences in their analyses. We found the Bacteroidaceae and Alcaligenaceae families were significantly lower in abundance in OVX compared to sham. Both of these families are associated with estrogen metabolism. Estrogens have been demonstrated to foster growth of Bacteroidaceae in the oral cavity 77 and Alcaligenaceae has been shown to metabolize estradiol in vitro 78. Our observations are consistent with this work, as reductions of these families are associated with reduced estrogen levels. Estrogen metabolites are directly exposed to the gut microbiome through enterohepatic circulation, but very little is known about the direct interactions between estrogens and the gut microbiome. Future investigations are required to determine whether there is a causal role of the gut microbiome in bone loss in OVX 30, 74, 79 and to explore specific mechanisms by which the gut microbiome may effect bone metabolism in sex‐steroid deficiency.

*The authors have declared no conflicts of interest*.

## Supporting information

Supplemental Figure 1. Regions of interest analyzed in the tibia using microCT.

Supplemental Figure 2. Within ‐group beta‐diversity is significantly different than the between‐ group diversity indicating that the composition of gut microbiomes from OVX rats significantly differ from those of sham rats (taxon abundance weighted and unweighted UniFrac; p<0.01, Bonferroni corrected non‐parametric t‐tests’).

Supporting Material
