# Supplementary figures and images for: Increasing dietary nitrate has no effect on cancellous bone loss or fecal microbiome in ovariectomized rats
Source: Mol Nutr Food Res. 2017 Mar 30;61(5):1600372. doi: 10.1002/mnfr.201600372 (PMC5434898; doi:10.1002/mnfr.201600372)

## Slide 1
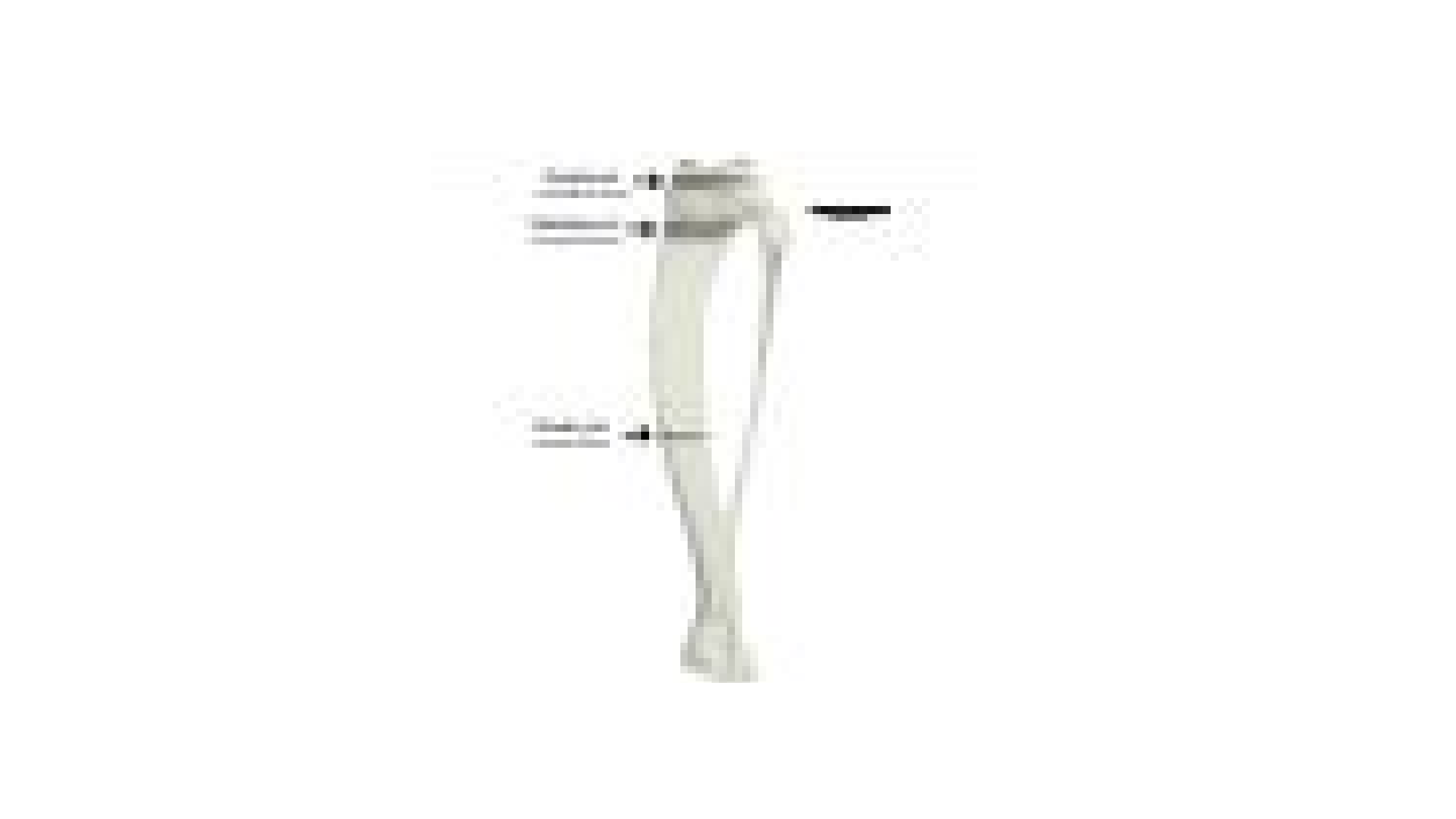

Supplement: Supplementary file 1 — Supplemental Figure 1. Regions of interest analyzed in the tibia using microCT. [file MNFR-61-na-s001.pptx]

## Slide 1
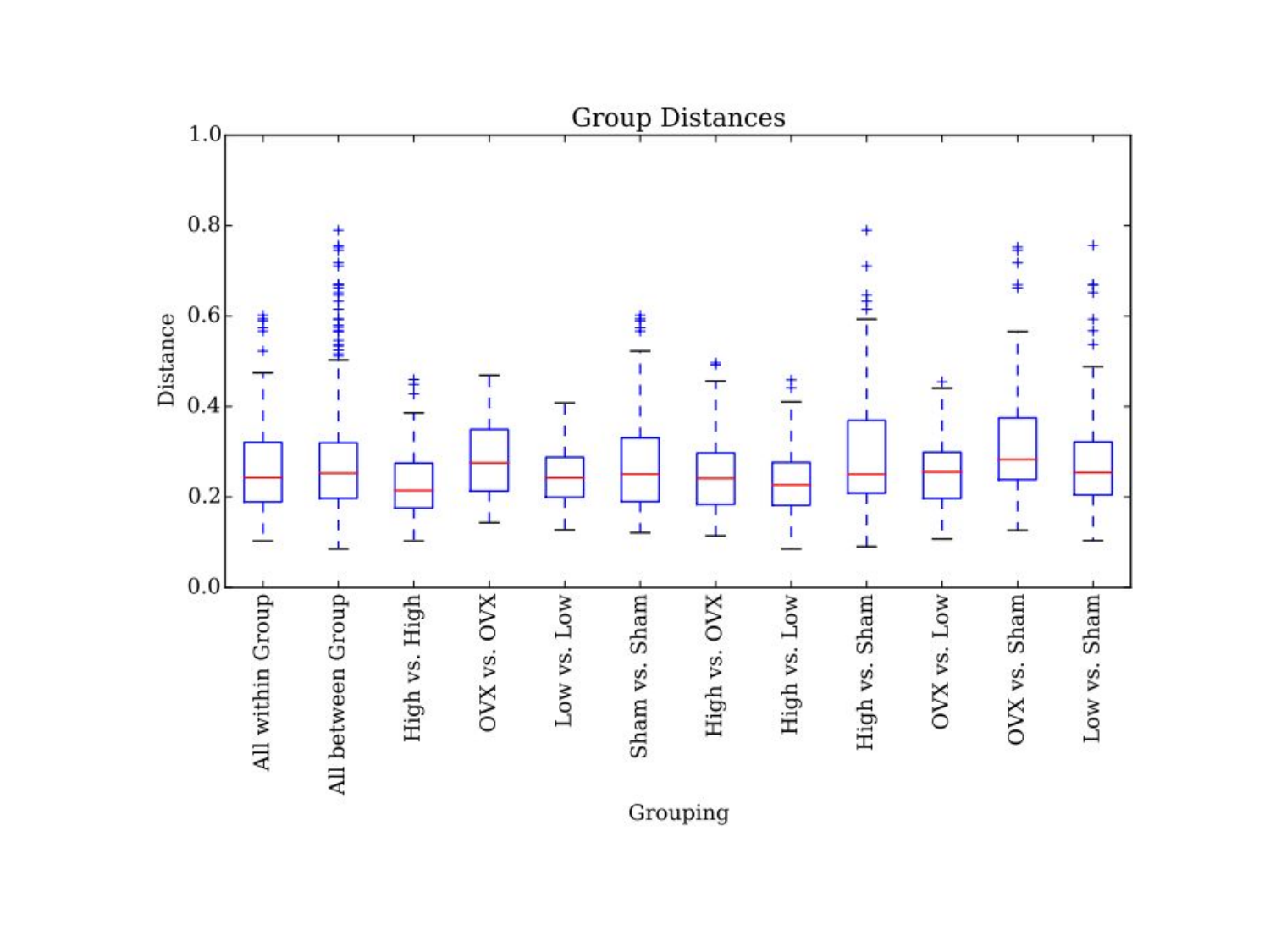

Supplement: Supplementary file 2 — Supplemental Figure 2. Within ‐group beta‐diversity is significantly different than the between‐ group diversity indicating that the composition of gut microbiomes from OVX rats significantly differ from those of sham rats (taxon abundance weighted and unweighted UniFrac; p<0.01, Bonferroni corrected non‐parametric t‐tests’). [file MNFR-61-na-s002.pptx]
