# Supplementary material for: Increasing dietary nitrate has no effect on cancellous bone loss or fecal microbiome in ovariectomized rats
Source: Mol Nutr Food Res. 2017 Mar 30;61(5):1600372. doi: 10.1002/mnfr.201600372 (PMC5434898; doi:10.1002/mnfr.201600372)
Supplement: Supplementary file 3 — Supporting Material [file MNFR-61-na-s003.docx]

**Histomorphometry**

Briefly, proximal tibiae were dehydrated in a graded series of ethanol and xylene and embedded un-decalcified in modified methyl methacrylate. Longitudinal sections (4 μm thick) were cut with a vertical bed microtome (Leica/Jung 2165) and affixed to slides pre-coated with a 1% gelatin solution. One section/rat was mounted unstained for measurement of fluorochrome labels. One section/rat was stained for tartrate resistant acid phosphatase and counterstained with toluidine blue (Sigma-Aldrich, St. Louis) for measurement of osteoclast perimeter.

The sampling site for the proximal tibia metaphysis was located 1mm distal to the growth plate and extended approximately 600μm in the marrow excluding cortical bone. Fluorochrome-based measurements of bone formation included: i) mineralizing perimeter (mineralizing perimeter/bone perimeter: cancellous bone perimeter covered with double plus half single label normalized to bone perimeter, %), ii) mineral apposition rate (the distance between two fluorochrome markers that comprise a double label divided by the 7 day inter-label interval, μm/day), and iii) bone formation rate (bone formation rate/bone perimeter: calculated by multiplying mineralizing perimeter by mineral apposition rate normalized to bone perimeter, μm^2^/μm/year). Osteoclast perimeter was determined as the percentage of cancellous bone perimeter covered by multinucleated (two or more nuclei) cells with acid phosphatase positive (red-stained) cytoplasm (osteoclast perimeter/bone perimeter, %). The sampling site for the proximal tibia metaphysis was located 1mm distal to the growth plate and extended approximately 1.2mm in the marrow excluding cortical bone. Different areas of interest (AOI) were used in fluorochrome and osteoclast analyses due to different magnifications necessary for analyses.

**Experimental design of effects of dietary nitrate supplementation on bone in growing rat model**

A total of 24 female 6-week-old Sprague Dawley rats were obtained from Charles River Laboratory (Hollister, CA). Three days after arrival, the animals were randomized by weight into a control (Group 1) and 3 treatment groups (Groups 2 through 4) (n = 6 per group). Rats in the control group were provided with TD.2018 chow (Teklad Lab Animal Diets (Madison, WI), 118/1.84 nmol of nitrate/nitrite per g), and distilled, deionized water. Rats in groups 2, 3, and 4 were fed with a purified low-nitrate TD.99366 chow (Teklad Lab Animal Diets, 16.5/1.5 nmol of nitrate/nitrite per g). Group 3 received sodium nitrate in distilled, deionized water to deliver an approximate dose of 0.7 mmol/kg BW/day. Group 4 received sodium nitrite in distilled, deionized water to deliver an approximate dose of 50 μmmol/kg BW/day. Food and water were provided *ad libitum* to all animals. The rats were housed individually and maintained on a 12-hour light: 12-hour dark cycle for the duration of study (one week). The Institutional Animal Care and Use Committee at Oregon State University approved the experimental protocol. Animals were maintained in accordance with the NIH Guide for the Care and Use of Laboratory Animals.

Food and water consumption and body weight were measured and water replaced every other day. for the duration of experiment. After one week of treatments, animals were fasted overnight and then sacrificed; animals were anesthetized with 2–3% isofluorane delivered in oxygen, and death was induced by exsanguination from the heart. Distal femurs were collected and snap frozen in liquid nitrogen for RNA analyses.

**Rat Osteoporosis RT² Profiler PCR Array**

RNA was extracted from distal femur using Trizol (Life Technologies) according to the manufacturer’s protocol. RNA was quantified via spectrophotometry using a nanodrop-1000. 1 µg of RNA was reverse transcribed in a 20ul reaction volume using a Qiagen kit. qRT-PCR was performed using the 7900HT fast machine from Applied Biosystems, according to the manufacturer’s protocol. Gene expression profiling of 84 genes in the distal femurs was performed using The Rat Osteoporosis RT² Profiler PCR Array (PARN-170ZE-4) (SuperArray, USA). Results were normalized to housekeeping genes in the array, B2m, Hprt1, and Ldha. Multiple tests were controlled for using Benajimini-Hochberg with a false discovery rate of 5%. Nitrate-deficient controls were defined as the control group that both nitrate and nitrite treatment groups were compared to. Statistics were performed with the RT2 Profiler™ PCR Array Data Analysis online software (<http://​www.​sabiosciences.​com/​pcrarraydataanal​ysis.​php#Excel)>

**References**

[1] Caporaso JG, Lauber CL, Walters WA, Berg-Lyons D, Huntley J, Fierer N, et al. Ultra-high-throughput microbial community analysis on the Illumina HiSeq and MiSeq platforms. ISME J 2012;6:1621–4. doi:10.1038/ismej.2012.8.

[2] Conley MN, Wong CP, Duyck KM, Hord N, Ho E, Sharpton TJ. Aging and serum MCP-1 are associated with gut microbiome composition in a murine model. PeerJ 2016;4:e1854. doi:10.7717/peerj.1854.
